# Pollen beetle offspring is more parasitized under moderate nitrogen fertilization of oilseed rape due to more attractive volatile signal

**DOI:** 10.1038/s41598-022-18030-0

**Published:** 2022-08-22

**Authors:** Valentina Zolotarjova, Triinu Remmel, Astrid Kännaste, Riina Kaasik, Ülo Niinemets, Eve Veromann

**Affiliations:** Institute of Agricultural and Environmental Sciences, Estonian University of Life Sciences, Fr. R. Kreutzwaldi 1, 51006 Tartu, Estonia

**Keywords:** Plant signalling, Agroecology

## Abstract

Biocontrol providing parasitoids can orientate according to volatile organic compounds (VOCs) of their host’s plants, the emission of which is potentially dependent on the availability of soil nitrogen (N). This paper aimed at finding the optimal N fertilization rate for oilseed rape (*Brassica napus* L.) to favor parasitism of pollen beetles (*Brassicogethes aeneus* Fab. syn. *Meligethes aeneus* Fab.) in a controlled environment. Pollen beetles preferred to oviposit into buds of plants growing under higher N fertilization, whereas their parasitoids favored moderate N fertilization. As a part of induced defense, the proportion of volatile products of glucosinolate pathway in the total oilseed rape VOC emission blend was increased. Our results suggest that the natural biological control of pollen beetle herbivory is best supported by moderate N fertilization rates.

## Introduction

Plants emit volatile organic compounds (VOC) in response to, and for the prevention of, herbivory. Such olfactory signals serve as cues for plant-associated insects to orient their behavior: plant odors emitted in response to herbivory can repel pests^1,2^ and attract natural enemies of herbivorous insects^2,3^ such as hymenopteran parasitoids^4–6^. Meanwhile, specialized herbivores often use the same cues to locate food plants^7^. The emission rates of VOC depend notably on growth conditions^8^, however, practical knowledge of VOC for parasitoid attraction via growth condition manipulation is still scarce^9^.

An abiotic factor easily manipulated by farmers is fertilization. Fertilization practices are often poorly justified and/or excessive^10,11^. The availability of nutrients can affect the emissions of various VOC in complex ways. Typically, the lack of a nutrient affects the production of volatiles indirectly^12^ via altering the overall plant physiological activity and the share of carbon allocation between primary and secondary metabolism^13^. Nitrogen (N) availability can increase^14^ or decrease^15^ emissions of certain volatiles, depending on plant species, growth stage and interaction with other environmental factors. In actively growing plants, high N availability speeds up primary metabolism and plant growth, while in plants with arrested growth improved nutrition can enhance storage and secondary metabolism^16,17^. Nitrogen is needed for the biosynthesis of glucosinolates (GLS), the anti-herbivore toxins found in the *Brassica* family, and their volatile derivatives isothiocyanates (emitted after tissue damage) mediate plant–insect interactions^18–20^. Since specialist herbivores can orient towards GLS^21^, high N in crops can even benefit pests^22–25^.

Oilseed rape (*Brassica napus* L.) has a relatively poor N use efficiency^26,27^ and to improve the yield^28,29^ is conventionally fertilized with high amounts of N that exceed 200 kg N ha^−1^^30,31^. *Brassica napus* is the most dominating oilseed crop in Europe^32^ and an important ‘break crop’ of many arable rotations: oilseed rape and turnip rape (*Brassica rapa* L.) yields in 2019 were almost 1.5 times larger than sunflower harvest. *Brassica napus* is mainly cultivated for its seed, which are crushed to extract oil. The oil is used for cooking, lubricant, soaps and also for biofuels^33^.

Oilseed rape is attacked by a broad variety of pests. These include insects, nematodes, slugs and wood pigeons (*Columbia palumbus*)^34^. Pollen beetle (*Brassicogethes aeneus* Fab. syn. *Meligethes aeneus* Fab) is the most numerous pest of oilseed rape in Northern countries like Estonia^35^. Oilseed rape is most susceptible to pollen beetles during the bud stage (BBCH 51–59)^36,37^ when adults destroy flower buds to feed on the pollen^38,39^. Subsequently, beetles oviposit into 2–3 mm long buds^40^, and finally the emerged larvae also feed on pollen^34^. Although adult pollen beetles are polyphagous and feed on pollen of flowering plants of different families before and after overwintering, they prefer to lay their eggs on *Brassica* spp^40,41^. *Brassica napus* is especially vulnerable due to greater bud availability at a time when other food plants of *B. aeneus* have already passed the bud stage^40^.

Insecticides are a widespread weapon against pollen beetles. According to the Integrated Pest Management (IPM) strategy (promoted by the European Parliament and the Council through directive 2009/128/EC), insecticide use has several specific demands before application: pest threshold monitoring, application time (e.g. plant growth stage, weather conditions, wind velocity etc.) and choice of appropriate quantities^42^. Despite this, pesticides are often applied routinely without regard of pest incidence and abundance^38^. This situation has caused the development of pollen beetles’ resistance to pyrethroids in Europe^42–44^. The use of insecticides not only pollutes the environment^38^, but also kills beneficial non-target arthropods such as pollinators and natural enemies of pests^34,38,42,45,46^.

Sustainable biocontrol of pollen beetles relies on naturally occurring parasitoids. Parasitoids lay eggs into *B. aeneus* eggs and/or larvae and the emerging parasitoid larvae feed and develop in the host, eventually killing it^47^. The effectiveness of parasitoids has been demonstrated in natural conditions by many field studies^35,48–53^ as well as in controlled environment^25^.

In this study, we investigated the effects of N fertilization on the infestation of *B. napus* by *B. aeneus* and the parasitism rate of *B. aeneus* in a controlled environment to gain a detailed insight into how fertilization affects insect host preference and prevalence of parasitism and how insect performance depends on volatile emission profiles of differently fertilized plants. The main aim of our study was to find the optimal N fertilization rate for *B. napus* to favor parasitism rate among pollen beetles while maintaining high yield.

Two types of experiments were conducted: (i) host selection (HS) by pollen beetles and their parasitoids to link the fertilization status of plants to insect behavior; and (ii) collection of volatile organic compounds (VOC) from plants to find the characteristic components of plant odors that are responsible for attracting or repelling insects. The VOC experiment was repeated in two consequent years, 2013 and 2014.

## Materials and methods

### Plant and insect material

For the experiments we used cultivated *Brassica napus* plants and a pest insect *Brassicogethes aeneus*, as well as three hymenopteran parasitoid genus infecting the pest (*Phradis* spp., T*ersilochus* spp. and *Diospilus* spp.). None of these genus belong to endangered species registers and their use in experiments is not regulated by IUCN Policy Statement on Research Involving Species at Risk of Extinction. Wild plants were not used. In the research we followed all the relevant guidelines and legislatures of the Estonian Code of Conduct for Research Integrity.

Winter oilseed rape plants, pollen beetles and their parasitoids were obtained in Tartu County, Estonia (Supplementary Table S1). Plants were collected from agricultural field (Table S1) after overwintering at an early growth stage (BBCH 30–35) to avoid any pest infestation; thereafter replanted into 5 L pots. Plant individuals for Host selection experiment were kept in growth chamber (type KRK20, Flohr Instruments, Netherlands) with the light intensity 180 μmol m^−2^ s^−1^ at plant level (15 h day length), temperature between 2 and 10 °C and with relative humidity 65%. Plants for the VOC experiment were stored with light intensity at plant surface 180 μmol m^−2^ s^−1^, 15 h day length and relative humidity 60% in a growth room in 2013 (temperature from 18 to 25 °C) and a growth chamber (type PLG 1000, Flohr Instruments, Netherlands) in 2014 (temperature adjusted to outdoor conditions between 2 and 25 °C). In 2013, the soil was a 1:1 mix of sand and loamy soil taken from the plant growth location. In 2014, the plants were replanted into commercial peat soil (Estonia Peat Products LTD, pH 5.6–6.4) (Table 1) to improve plant nutrition and avoid pathogens naturally occurring in the field soil. In both years, plants (green/yellow bud stage, BBCH 53–59) were fertilized once on 8th of May with different levels of ammonium nitrate (NH_4_NO_3_) adjusted to soil surface area of each pot: 3 different N treatments in 2013 and 4 different treatments in 2014 (Table 1).

**Table 1:** Soil pH and nutrient concentrations (before fertilization) and N fertilization rates.

Year	pH	NO_3_-N mg/kg	NH_4_-N mg/kg	P mg/kg
**Soil**				
2013	7.13*	5.1 ± 0.6*	0.02 ± 0.01*	122.5 ± 3.5*
2014	5.6–6.4	302 ± 99.7	215 ± 71.0	603 ± 199.0

**Nitrogen fertilization**				
Ammonium nitrate (NH_4_NO_3_), kg ha^−1^ (number of plant individuals)				
2013 VOC	0 (6)	20 (10)	120 (7)	
2014 VOC	0 (2)	80 (3)	100 (3)	160 (3)
2014 HS	0 (21)	80 (19)	100 (19)	160 (20)

The number of plant replicates measured at each N fertilization treatment is in brackets. Field soil in 2013 contains autumn and spring soil content measurements by lactate extractable method for phosphorus with standard errors. Commercial peat soil in 2014 represents nutrient content from manufactory (as standard deviation was taken dry mass by capacity range).

*values before mixing with sand.

### Host selection experiment

Beetle and parasitoid host selection experiment (HS) was conducted in a walk-in (3 × 3 m) growth chamber (type KRK20, Flohr Instruments, Netherlands). Light intensity was 180 μmol m^−2^ s^−1^ at plant level (16 h day length), relative humidity fluctuated with watering in a range between 65 and 90% and the temperature was changed according to outdoor conditions from 10 to 25 °C (the experiment lasted 3 months). As N fertilization affects the odor of *B. napus* individuals^50^, plants were placed into groups (allocation in Supplementary Fig. S1a) with the intent to prevent the contamination of VOC signals by neighboring plants with different N treatments.

Five days after N fertilization the plants BBCH (55–62) was recorded and beetles were released uniformly (24 jars opened simultaneously) (Fig. S1a) into the growth chamber to select an oilseed rape plant for feeding and oviposition. In total 240 beetles (kept without food for 24 h) were released (three times the number of plants in the growth chamber) simulating the economic threshold densities (pesticide costs lower than the cost of potential yield loss) for winter oilseed rape in Scandinavia and Baltic Countries (1–2 beetles per plant in the bud stage and four just before flowering). All beetles and open flowers found on each plant were counted after 3, 6, 24, 48 and 72 h from the release of beetles. When pollen beetle parasitoids appeared outdoors on *B. napus* agricultural fields (21 days after the release of pollen beetles into the growth chamber), 26 adults collected from the field same day were introduced to the chamber (Table S1, Fig. S1a). Before releasing, we checked the sex of parasitoids to be sure that there are female individuals as well but we did not count them and released them as quickly as possible to minimize their stress.

Four weeks after the beetle introduction, water and sticky paper traps were installed to catch beetle larvae as they drop to the ground for pupation. A water trap consisted of a folium plate (30 cm length, 26 cm wide, 2.5 cm high) filled with water. The pot with *B. napus* was placed in the center of the water trap. After the installation of traps, the plants from different fertilization groups were rearranged inside the growth chamber breaking up the groups (Fig. S1b) to prevent the larvae from migrating between the plants. Water traps were refilled when needed. Metal hoops (100 × 30 × 0.5 cm, Tarha) and polypropylene twine (Horticom) were used to secure plant branches within water trap range. Sticky paper traps (AB “Insect Control—Greenhouse”, Silvandersson, Sweden) with glue on both sides, were placed on top of the soil on each pot. Water traps were emptied weekly, the upper sides of the sticky traps once every two weeks, and the bottom sides once at the end of the experiment. The insects were collected for six weeks.

To determine the parasitism rate, all collected pollen beetle larvae were dissected. All parasitoid larvae and eggs found were counted and identified^47^ to species level if possible. The parasitism rate of the beetle larvae was determined for each plant.

After the collection of beetle larvae, the remaining open flowers on each plant were counted. All developed siliques were collected, counted and dried outdoors in paper bags for a week (daytime temperature 25 °C). Dried seeds were manually taken from siliques, weighed and counted automatically with a seed counter (Automatic Seed Counter Machine SLY-C, China).

### Volatile organic compound (VOC) measurements

#### VOC collection

The upper parts of *B. napus* plants were enclosed individually in airtight glass chambers (3 L)^54^. A small fan in every chamber provided uniform gas concentrations. Four chambers were operated simultaneously having one unique treatment member per chamber; the chamber for each treatment group was chosen randomly avoiding same fertilization representative in the same chamber during next round of measurement. During the experiment, air temperature 28 °C and light intensity at plant surface 200 μmol m^−2^ s^−1^ was provided by two halogen lamps (300 W, Bemko). After the experiment, area of leaves and flower number in the chamber during the experiment were estimated.

During each VOC collection, 4 L of air was pumped 20 min through an adsorbent with an air sampling pump (224-PCXR8, Airchek Sampler, SKC Inc., USA). The cartridges were filled with three different carbon-based adsorbents to quantitatively adsorb all volatiles in C3–C17 range^55^. The chamber inlet air was purified with a charcoal filter (Scrubber Assy 4291, Thermo Electron Corporation).

VOCs were collected at June in 2013 and at the end of May in 2014. Plants used in the experiment had growth stages from flower buds only to already flowering (BBCH 57–65). Blank air samples were collected from each empty chamber before inserting the plants. The collections were carried out in groups of four plants (one from each treatment group, randomly placed), two groups per day, one starting at 12:00 and the other at 16:00. The plants placed into the chamber system were allowed to acclimate for half an hour before VOC collected. Thereafter, four *B. aeneus* individuals (starved for 24 h beforehand) were placed on each plant, the plants were left to stabilize for an hour and a new collection of VOCs was conducted. As a result, VOCs were collected from each plant before and after the release of pollen beetles. In order to prevent beetles escaping (and damage due to the fan), they were enclosed in a small transparent nylon organza bag (8.5 × 12 cm) surrounding the main raceme of each plant. Each chamber had one and the same bag during all measurements (including the blanks).

#### VOC Identification and calculation of emission rates

The cartridges were desorbed and volatiles analysed by a combined Shimadzu TD20 automated cartridge desorber and Shimadzu 2010 Plus GC–MS system (Shimadzu Corporation, Kyoto, Japan)^56^. VOC were identified based on authentic standards and comparison of the mass spectra with NIST library (National Institute of Standards and Technology, 2014). Part of the chromatograms (from 42% of all plant individuals in both years) were investigated manually to develop a custom-made mass spectral library (plant VOC only) for automatic processing with the open source software OpenChrom (Community Edition, ver. 1.2.0, Alder, lablicate.com/platform/openchrom). The following procedures and settings were used in Open Chrom: background noise reduction, AMDIS peak detection (S/N ratio = 2, medium threshold and high shape requirement), trapezoid peak integration and compound identification with 80% similarity match factor. All chromatograms were subsequently checked for false positive/negative compound identification. Empty chamber VOC concentrations were subtracted from the values with the plant and volatile emission rates were calculated^57^. Adsorbed air was analysed for Geranyl diphosphate (GDP), Geranylgeranyl diphosphate (GGDP), Lipoxygenase (LOX), Glucosinolate (GLS) and Shikimate (SHI) pathways VOC, where emissions were expressed per unit leaf area^56,58^.

### Statistics

The influence of N fertilization on *B. napus* yield (siliques and seeds dry masses precision of 1 × 10^−4^ g)*,* the abundance of *B. aeneus* and its parasitoids and flowers and at the harvest time were tested with One-way ANOVA (ANCOVA for parasitized larval number) type III, Fisher Least Significant Difference for Post Hoc comparison and Pearson correlation using STATISTICA 12 (Statistica 64, version 13.2 Dell Inc.). Plant observations in 0, 3, 6, 24, 48 and 72 h after the beetle release were examined with same software utilizing Repeated ANOVA t. III. All significant correlation results passed the Bonferroni correction.

VOC emissions were Log-transformed to normalize the distributions for parametrical tests. Using STATISTICA 12 the data was tested with Repeated ANOVA t. III, One-way ANOVA t. III and Spearman correlation for N-fertilization and herbivory investigation of total emission, compounds biosynthetic pathways and each compound separately. The relationships between plant growth stage, the number of flowers in the chamber during VOC collection and fertilization rate were tested with Spearman correlations. To test VOC influence on parasitoids and their offspring, an average emission from 2014 VOC experiment was applied on HS plant data (Log-transformed) and tested with Pearson correlation.

VOC and parasitoid species assemblages were visualized with non-metric multidimensional scaling (NMS). Found pattern significance was tested with Multi-Response Permutation Procedure (MRPP) using PC-ORD (ver. 6.19; McCune and Mefford 2011). Each ordination was tested at least 5 times^59^, stress factor < 19 and the number of randomizations was 250 for NMS. The distance measures of Sørensen (Bray–Curtis) for VOC data. Since in VOC analyses the data of the same plant individual was frequently utilized in the same test twice (non-infested by pollen beetle and infested), general relativization by compound (eg division by compound maximum value) was applied to avoid potential pseudo replication.

Treatment effects were considered statistically significant at *p* < 0.05. All data was checked for normality of distributions by Kolmogorov–Smirnov test before applying parametric tests.

## Results

### N effects on plant traits

Yield components depended significantly on fertilization rate (Table 2). There were no differences in the number of flowers between fertilization groups measured during three days after pollen beetle infestation (*p* > 0.9), or at the harvest time (Table 2). The number of remaining flowers during harvest was negatively correlated with the number (*r* = − 0.518, *n* = 79, *p* < 0.001) and mass (*r* = − 0.389, *n* = 79, *p* < 0.001) of seeds along with the number of siliques per plant (*r* = − 0.536, *n* = 79, *p* < 0.001). Seed number and mass per plant correlated as well (*r* = 0.860, *n* = 79, *p* < 0.001).

**Table 2:** One-way ANOVA and Pearson correlation test results for the effects of nitrogen fertilization on *Brassica napus* yield and *Brassicogethes aeneus* larval abundance and their parasitized number.

Per plant	Pearson correlation	One-way ANOVA	N0	N80	N100	N160
Silique number	*r* = 0.396	*F*_(3,75)_ = 5.565	3191^a^	3994^b^	4171^b^	4487^b^
	*p* < 0.001	*p* = 0.002	*152* ± *11*	*210* ± *11*	*220* ± *9*	*224* ± *22*
Seed number	*r* = 0.284	*F*_(3,75)_ = 3.113	28,436^a^	34,204^b^	35,239^b^	36,206^b^
	*p* = 0.011	*p* = 0.031	*1354* ± *119*	*1800* ± *126*	*1855* ± *93*	*1810* ± *185*
Seed mass (g)	*r* = 0.197	*F*_(3,75)_ = 2.100	152^a^	183^b^	183^bc^	181^abc^
	*p* = 0.081	*p* = 0.012	*7* ± *1*	*10* ± *1*	10 ± 1	9 ± 1
Flower number	*r* = 0.036	*F*_(3,75)_ = 0.200	133^a^	93^a^	74^a^	164^a^
	*p* = 0.751	*p* = 0.899	*6* ± *3*	*5* ± *3*	*4* ± *2*	*8* ± *5*
Larval number	*r* = 0.180	*F*_(3,75)_ = 2.455	219^a^	245^abc^	167^ab^	326^c^
	*p* = 0.113	*p* = 0.070	*10.4* ± *2.5*	*12.9* ± *2.3*	*8.8* ± *1.6*	*16.3* ± *2.3*
Parasitized larval number*	*r* = 0.045	*F*_(3,74)_ = 4.004	14^a^	37^b^	21^abc^	17^ac^
	*p* = 0.697	*p* = 0.011	*0.7* ± *0.1*	*2.0* ± *0.6*	*1.1* ± *0.3*	*0.9* ± *0.3*

Plant traits were calculated at harvest date and insects represent cumulative data up to the harvest. Nitrogen impact on the number of parasitized *B. aeneus* larvae was tested with ANCOVA. Significantly distinct (*p* < 0.05) fertilization groups by Fisher LSD (Least Significant Difference) marked with different letters (Post Hoc comparison).

N0-N160—fertilization treatments (kg ha^-1^ N).

The number of replicates in each treatment was: N0 = 21, N80 = 19, N100 = 19, N160 = 20.

*r* represents Pearson correlation coefficients (*n* = 79 plants).

The average values and standard errors are in italics.

*Total number of larvae was taken as a covariate.

Seed number per silique was weakly correlated to N fertilization level (*r* = − 0.251; *p* = 0.026), the highest value (i.e. most seeds per one silique) was found in untreated plants. Silique number per plant correlated with seed number (*r* = 0.791, *n* = 79, *p* < 0.001) and seed mass per plant (*r* = 0.670, *n* = 79, *p* < 0.001).

### Pest abundance effects on yield

**Figure 1:**
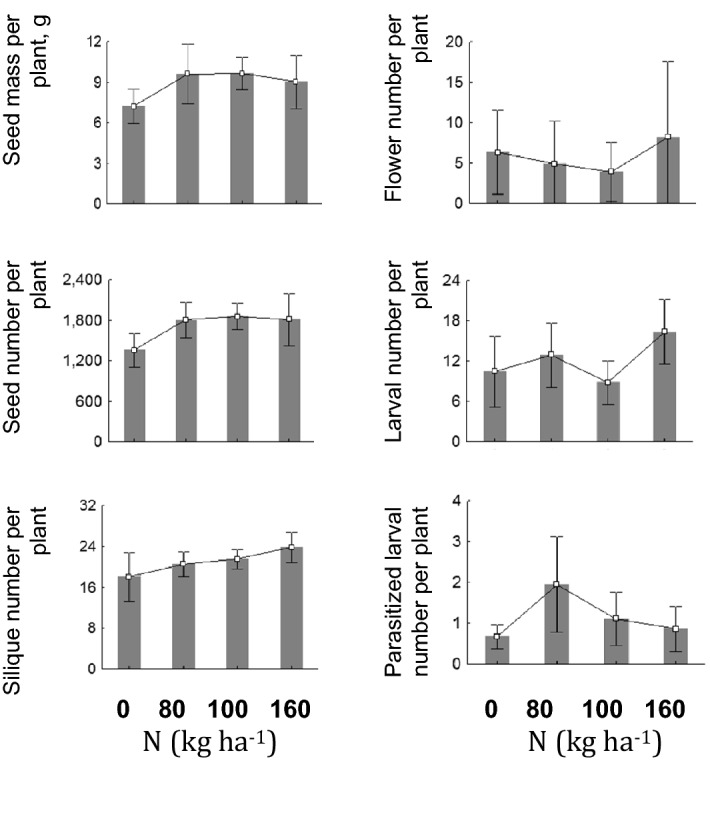
*Brassica napus* yield and the numbers of flowers (on harvesting day of HS experiment, end of July 2014), beetle larvae and parasitized larvae in dependence on the fertilization rate (means ± 95% confidence intervals). N—nitrogen fertilization rate (0, 80, 100, 160 kg N ha^−1^).

Although seed mass per plant was not linearly correlated with additional nitrogen (Fig. 1), it was in negative linear dependence on *B. aeneus* larval number per plant (*r* = − 0.242, *n* = 79, *p* = 0.032).

The abundance of pollen beetle larvae was correlated to the number of parasitoids (*r* = 0.540, *n* = 79, *p* < 0.001), however the latter has no significant correlation with other yield traits.

### N and year effects on VOC emission

In total, 32 different VOCs were identified in both years (Tables S2–S3). Nitrogen fertilization (*A* = 0.075, *p* < 0.001) and the year of experiment (*A* = 0.075, *p* < 0.001) had an impact on plant VOC pathways conforming to MRPP test. Half of all N groups were not distinct from each other in MRPP pairwise comparisons (Fig. S2a under the graph).

According to MRPP test volatile compounds were influenced by N fertilization (*A* = 0.073, *p* < 0.001) and by year (*A* = 0.045, *p* < 0.001) (Fig. S2b). Few N groups were not distinct from each other in MRPP pairwise comparisons (Fig. S2b under the graph). VOC were correlated with the number of flowers and BBCH at the period of beetle release (both *r*^2^ > 0.1).

**Figure 2:**
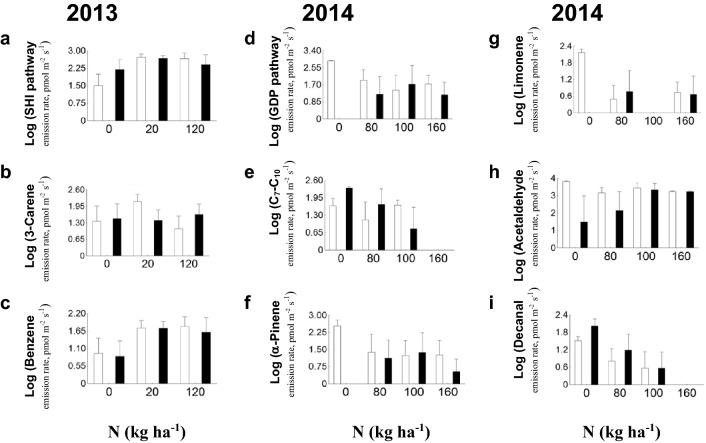
Emission rates (mean + SE) of different VOC formation pathways (**a**, **d**, **e**) and individual compounds (**b**, **c**, **f**, **g**, **h**, **i**) from *Brassica napus* plants in 2013 and 2014 in relation to nitrogen fertilization before (white bars) and during pollen beetle infestation (black bars). SHI—shikimate pathway volatiles; GDP—geranyl diphosphate pathway volatiles; C_7_–C_10_—long-chained saturated aldehydes.

In 2013, there was no significant N fertilization impact on LOX, GDP, GGDP and GLS pathway compounds as well as total VOC. Only SHI pathway products were affected by additional N (*F*_2,20_ = 4.028; *p* = 0.034), with lower emissions for unfertilized plants (Table S2, Fig. 2a).

In 2014, fertilization had no impact on LOX, GGDP, GLS and SHI pathway products as well as total VOC. The number of flowers VOC was collected from was negatively correlated with fertilization rate (*r*_s_ = − 0.623; *n* = 11, *p* = 0.041), but not with growth stage of *B. napus* (*r*_*s*_ = − 0.550; *n* = 11, *p* = 0.079) in 2014.

### Beetle effects on VOC emission

The smell of *B. napus* did not significantly change (MRPP test: *A* = − 0.009, *p* = 1, Fig. S2a and *A* = 0.008, *p* = 1, Fig. S2b centroids adjoin in NMS ordination) with beetle presence (both years). However, GLS pathway products share from total emission increased by 3% in 2013 and by 42% in 2014 with beetle infestation. VOC emissions from different pathways were associated with flowers and BBCH during infestation with beetles (Fig. S2a).

In 2013 among individual LOX representatives 2-pentanone (*F*_2,20_ = 3.619; *p* = 0.046) has been affected by fertilization before infestation with beetles (less emission from N0 individuals, Table S2) but not after. In the same year GDP pathway compound 3-carene (*F*_2,20_ = 3.857; *p* = 0.038) emission increased after infestation for N0 and N120 but decreased for N20 plants (Table S2, Fig. 2b). Among SHI pathway members benzaldehyde emission moderately increased along the fertilization gradient (*r*_*s*_ = 0.443, *n* = 23, *p* = 0.034) before beetle appearance and fertilized plants had higher emission rate of benzene (*F*_2,20_ = 4.503; *p* = 0.024) in 2013 (Table S2, Fig. 2c). Infested plants emissions of acetaldehyde (*r*_*s*_ = 0.44, *n* = 23, *p* = 0.036) and acetone (*r*_*s*_ = 0.541, *n* = 23, *p* = 0.008) were enhanced by additional nitrogen in 2013 (Table S2). In the same year the number of flowers (inside chamber during VOC collection) and the growth stage of *B. napus* did not correlate with fertilization rate.

In 2014 GDP pathway products emission decreased after *B. aeneus* appearance (*F*_1,7_ = 8.455, *p* = 0.023; Table S3, Fig. 2d). In the same year long-chained saturated aldehydes had strong negative correlation with nitrogen fertilization during beetle activity (*r*_*s*_ = − 0.713, *n* = 11, *p* = 0.014) but not before (Table S3, Fig. 2e). Among individual GDP volatiles in 2014, emissions of α-pinene were significantly reduced as a reaction to the beetles (*F*_1,7_ = 9.062; *p* = 0.020; Fig. 2f), whereas limonene emission was reduced for control and intensive nitrogen groups, while plants with moderate fertilization—N80—showed an increase (*F*_3,7_ = 5.798; *p* = 0.026; Table S3, Fig. 2g). The LOX pathway product 2-pentanone (*F*_3,7_ = 6.246, *p* = 0.022) as well as GLS pathway product isocyanatocyclohexane (*F*_3,7_ = 4.425, *p* = 0.048) was affected by N fertilization in uninfested plants (2014) as follows: there was no emission in N80 plants, while the highest values were in N100 plants (Table S3). Beetle activity reduced the emissions of the short-chained oxygenated compound acetaldehyde (*F*_1,7_ = 5.836; *p* = 0.046; Table S3, Fig. 2h). Among long-chained saturated aldehydes, decanal emission was negatively correlated with fertilization rate both before the infestation with *B. aeneus* (*r*_*s*_ = − 0.613, *n* = 11, *p* = 0.045) and after (*r*_*s*_ = − 0.845, *n* = 11, *p* = 0.001) (Table S3, Fig. 2i), with higher emission after infestation. Oilseed rape top part (the VOC was collected from) flowers were negatively correlated with fertilization rate (*r*_s_ = − 0.623; *n* = 11, *p* = 0.041), but not with growth stage of *B. napus* (*r*_*s*_ = − 0.550; *n* = 11, *p* = 0.079) in 2014. Several individual compounds were positively or negatively correlated with the number of beetles and/or parasitoids (Table 3), indicating potential attractants and repellents for these insects.

**Table 3:** Pearson correlation test results of VOC emission (LOG-transformed) of pollen beetle larvae, parasitoid offspring and parasitized larvae of pollen beetle (count of unique individuals despite of single- or multi-infestation).

Positive correlation		Negative correlation	
**Oilseed rape (n = 79) VOC with pollen beetle larvae**			
6-Methyl-5-hepten-2-one	*r* = 0.236, *p* = 0.036	Acetaldehyde	*r* = − 0.248, *p* = 0.028
		Decanal	*r* = − 0.268, *p* = 0.017
		Toluene	*r* = − 0.263, *p* = 0.019
**Oilseed rape (n = 19) VOC with pollen beetle parasitoid offsprings**			
*(Z)-*3-Hexen-1-ol	*r* = 0.720, *p* = 0.001	2-Propenenitrile	*r* = − 0.638, *p* = 0.003
$\alpha$-Pinene	*r* = 0.531, *p* = 0.019	Acetone	*r* = − 0.649, *p* = 0.003
Benzothiazole	*r* = 0.628, *p* = 0.004	Acetophenone	*r* = − 0.613, *p* = 0.005
Limonene	*r* = 0.501, *p* = 0.029	Isocyanatocyclohexane	*r* = − 0.727, *p* < 0.001
Heptanal	*r* = 0.480, *p* = 0.038	Hexane	*r* = − 0.675, *p* = 0.002
Octanal	*r* = 0.528, *p* = 0.020	Methanethiol	*r* = − 0.615, *p* = 0.005
3-Carene	*r* = 0.539, *p* = 0.017		
**Oilseed rape (n = 19) VOC with parasitized larvae of pollen beetle**			
*(Z)-*3-Hexen-1-ol	*r* = 0.542, *p* = 0.017	Isocyanatocyclohexane	*r* = − 0.524, *p* = 0.021
$\alpha$-Pinene	*r* = 0.466, *p* = 0.044	Hexane	*r* = − 0.514, *p* = 0.025
Benzothiazole	*r* = 0.536, *p* = 0.018		
Octanal	*r* = 0.480, *p* = 0.037		
3-Carene	*r* = 0.486, *p* = 0.035		

Untreated plants VOCs were used for the correlation test with pollen beetle larvae and infested plants VOCs for correlation test with beetle parasitoid offspring and parasitized larvae of pollen beetle.

### N effects on pest and parasitoid preferences

In total, 957 *B. aeneus* larvae were collected, 90.7% (868) were uninfested by parasitoids, 7.1% (68) had one parasitoid and 2.2% (21) had more than one. Five parasitoid species were found, three of which were identified to the species level (Table 4). The most abundant parasitoid species found in *B. aeneus* larvae was *Diospilus capito* Nees, followed by *Tersilochus heterocerus* Thomson. The highest number of *B. aeneus* larvae was found on N160 plants and the lowest on N100 while greatest parasitism rate was discovered on N80 plants and the lowest on N160 (Table 4). The choice of food plant of pollen beetles did not depend on N treatment (*p* > 0.2) during the first three days and at the end of experiment (Table 2) except for Post Hoc comparison where N160 treatment differed from unfertilized and N100 plants.

**Table 4:** Total and average ± SE number of *Brassicogethes aeneus* larvae parasitism rates and the total numbers of parasitoid offspring species collected from oilseed rape plants under different nitrogen (N) treatments.

N fertilization rate (kg ha^-1^)	N0		N80		N100		N160		Total
Parasitoids	18	*0.9* ± *0.2*	54	*2.8* ± *0.7*	23	*1.2* ± *0.4*	21	*1.1* ± *0.3*	116
Parasitation rate (%)	6.4	*7.1* ± *1.8*	15.1	*11.0* ± *2.4*	12.6	*13.9* ± *3.4*	5.2	*4.5* ± *1.5*	9.3
*Diospilus capito*	7	*0.3* ± *0.1*	34	*1.8* ± *0.5*	15	*0.8* ± *0.3*	9	*0.5* ± *0.2*	65
*Phradis morinellus*	1	*0.05* ± *0.1*	–	*–*	1	*0.1* ± *0.1*	–	*–*	2
*Sp1*	2	*0.1* ± *0.1*	–	*–*	1	*0.1* ± *0.1*	1	*0.1* ± *0.1*	4
*Sp2*	–	–	1	*0.1* ± *0.1*	–	*–*	–	*–*	1
*Tersilochus heterocerus*	8	*0.4* ± *0.1*	19	*1.0* ± *0.4*	6	*0.3* ± *0.2*	11	*0.6* ± *0.2*	44

The average values and standard errors are in italics.

Parasitized larvae number per plant depended on N fertilization (Table 2) but not on parasitoid species according to the MRPP test (*A* = 0.025 *p* = 0.07). However, N0 and N80 groups were distinct (*A* = 0.05, *p* = 0.019) in pairwise comparison.

## Discussion

### N effects on plant traits

Nitrogen fertilization affected the yield of oilseed rape (Table 2). Total mass and number of seeds per plant did not differ much between fertilized plants, but all these groups had higher yields than N0 (Fig. 1). Similar results were found in other studies^50,60–62^.

Fertilization with N had a clear positive effect on the number of siliques (Table 2, Fig. 1) as in open field conditions^28,50^ and there was a weak negative correlation on seed number per silique. The trend of decreased number of seeds per siliques with higher N-fertilization may reflect low nitrogen use efficiency^63^.

### Pest abundance effects on yield

The relationship between yield and pest abundance was not linear in our study (Fig. 1). Although N160 plants had the highest pollen beetle infestation, this did not in general decrease the yield of N160 plants. Pest damage might have caused earlier silique formation^64,65^ and since adult pollen beetles do not feed on seeds, this may imply the pest resistance mechanism called avoidance^66^. As a result, these plants had the highest silique quantity (Fig. 1).

On the other hand, oilseed rape plants can respond to the floral bud losses caused by pollen stealing by *B. aeneus* with another well-developed compensation strategy. Compensation is usually achieved by the production of new floral buds^67^ on already existing or newly developed branches^68^. The formation of new branches and inflorescences compensates for floral bud losses and finally, may also increase the ratio of seed to total plant mass, increasing crop productivity^69^. Therefore, despite distinct amount of pollen beetle larvae in zero and highest fertilization treatments, seed mass per plant was similar for mentioned groups^70^(Table 2).

### N and year effects on VOC emission

Nitrogen fertilization impacted VOCs emission differently between experimental years. In 2013, SHI pathway compound emissions (especially benzene and benzaldehyde) from fertilized plants were higher compared to unfertilized, regardless of herbivory due to better nitrogen supply^71^. In the latter research the concentration of SHI compound benzoic acid increased with N fertilization due to primary and secondary metabolites competition reduction. In infested plants, fertilization rate was positively correlated with acetone and acetaldehyde (short-chained oxygenated volatiles) production. This may be ecologically significant, since both of these compounds are considered to be hazardous VOCs and can be emitted by plants in substantial quantities in certain environmental stress conditions^72^. On the other hand, high background emissions of these compounds can reflect higher activity of certain metabolic pathways such as isoprenoid or lipid metabolism^73^, which is plausible in more actively growing plants at higher level of fertilization. Moderately fertilized plants (N20 in 2013 and N80, N100 in 2014) frequently showed different results in our study, most probably because these plants were optimally fertilized, as opposed to others that were under or over fertilized with different nutrients^74,75^.

In 2014, a strong negative correlation with N-fertilization was revealed, such that no decanal emission was detected in N160. A similar trend was found same year for the whole class of long-chained saturated aldehydes (C_7_–C_10_), with no detected emission in N160 and negative correlation with N-fertilization during beetle activity. Since the number of flowers during VOC collection was negatively correlated with N fertilization, we suggest that despite decanal and other long-chained saturated aldehydes, decanal, heptanal, nonanal and octanal, are commonly emitted from plant leaves^56,76,77^, they can be a part of flower scent. For example, these aldehydes were components of flower scent in hawthorn (*Crataegus monogyna*) and raspberry (*Rubus idaeus*)^78^.

There were various quantitative and qualitative differences in the plant VOCs measured in 2013 vs 2014. Since the nutritional status of the plants was not evaluated and we used commercial soil in 2014 (field soil in 2013) we can speculate that VOC discrepancy occurred mainly due to soil quality variation between experimental years^8^. Since the *B. napus* varieties that we used emitted almost the same array of compounds, but with different relative proportions, similarly to other study^79^, we assume that the varieties along with different N-supply play a part in variances between the years.

### Beetle effects on VOC emission

Pollen beetles affected the scent of oilseed rape. The proportion of GLS in the total emission increased in both experimental years after beetle appearance, more GDP pathway compounds were detected (emission is higher than zero) before beetle release (both years) and 2-pentanone (LOX) emission increased in 2014 (except for N100) after the infestation with beetles. GLS and LOX pathway compounds take part in induced defense against herbivores as toxins and repellents; moreover, GLS is specific to *Brassicaceae*^18,19,80–82^. Herbivores ability to sequester toxins repels their enemies^22^ explaining the negative correlations of GLS pathway (2-propenenitrile, isocyanatocyclohexane, maethanethiol) and LOX pathway (hexane) compounds with the number of pollen beetle parasitoid offspring found in our study. In addition, pollen beetles elicited the emission of GDP pathway compound 3-carene (except N20) in 2013, as in other research^50^ and reduced the emissions of α-pinene (except N100) and limonene (except N80) as well as total GDP emission in 2014. GDP pathway compounds are known to attract herbivores^50,82^; their reduction can therefore serve as induced defense, or olfactory camouflage. Pollen beetle parasitation in our study was positively correlated with (*Z*)-3-hexen-1-ol, α-pinene, limonene, benzothiazole, octanal and 3-carene emissions. Said compounds instigate herbivores oviposition^50,83–85^ and therefore may attract parasitoids.

Acetaldehyde served as pollen beetle repellent (Table 3) for uninfested *B. napus* and the infestation in 2014 provoked a reduction of acetaldehyde emission (Fig. 2h) for N0 and N80. In wooden species, acetaldehyde has been found to increase in response to herbivory^86,87^. However, there is a substantial variation among plant families in the mechanisms of acetaldehyde synthesis^88^, and different stress responses are therefore expectable. The presence of herbivores somewhat increased decanal emission in 2014 (Fig. 2i). Since decanal is negatively correlated with pollen beetle larvae in our experiment for uninfested plants, we can speculate it signals pollen beetle females to avoid oviposition and the same applies to toluene (Table 3). Pollen beetle larvae were attracted by 6-methyl–5–hepten–2–one (Table 3) which was found to also attract cattle flies^89^.

There is contrasting evidence about the total VOC response to herbivory in *Brassica* spp. In our experiments, the total emissions did not change after herbivory treatment, but an increase has been reported for cabbage (*Brassica oleracea* var. *gemmifera*)^90^ and a decrease for *Brassica nigra* infested with *Pieris brassicae* larvae^65^. Discrepancy in our experiment might be explained by different measurement times after herbivory, eg VOC were collected within 9 h in^90^ and 1.5 h in^65^. In our experiment VOC were collected 1 h after beetle release, consequently we can speculate that within 1 h *B. napus* total emission either does not change or could have recovered so that no difference was detected. But this hypothesis needs further investigation.

### N effects on pest and parasitoids preference

Nitrogen fertilization affected the oviposition choices of pollen beetle (N160 vs unfertilized and N100 plants) and their parasitoids (Table 2). In our study at 1, 3, 6, 24, 48 and 72 h after infestation, the number of pollen beetles per plant did not differ between treatment groups. We can speculate that the beetles had initially low host plant selectivity due to food deprivation (beetles starved 24 h before the experiment). That could explain why host plant quality did not play a role during the first 72 h, but its importance increased with time based on larval numbers at different N treatments (Fig. 1). In addition, it may also mean that pollen beetles were not selective when feeding but plant quality played a role in the selection of oviposition sites.

Beetles were most abundant at the highest N level although not significantly distinct. The increase of pollen beetle larval abundance along the N gradient most probably reflects host plant quality improvement for the pests, which positively influences their fecundity^22^. The preference of N160 plants for oviposition by females could be related to chemical cues that insects catch with antennae on bud surface during host selection or to differences in bud size: *B. aeneus* females strongly prefer to oviposit into 3–5.5 mm *B. napus* buds^91^. Consequently, we can speculate that better nutrition intensified *B. napus* growth in the N160 group, and optimal size buds appeared earlier, attracting more females (Fig. 1). Another possible explanation is a longer period of suitable buds due to compensation strategy of floral bud losses ^67^ caused by herbivory. However, bud size was not measured in this study and no clear conclusions concerning its importance can be made.

Parasitism rate was the greatest at intermediate fertilization levels. The study of tritrophic interaction on another *B. napus* pest *P. xylostella*^25^ has shown similar results, where low nitrogen levels in leaf tissues enhance parasitoid growth rate and total number of offspring and reduce the length of pupal stage^25^. Parasitoid preference for N80 may also be related to N-dependent production of volatile organic compounds, which helps insects navigate in host selection^5^. In such case, parasitoids will not navigate towards a higher number of beetle larvae, but towards a more attractive odor bouquet. Our study revealed that the smell of *(z)-*3-hexen-1-ol, $\alpha$-pinene, benzothiazole, octanal and 3-carene potentially attract and isocyanatocyclohexane and hexane repel pollen beetle parasitoids. On the contrary, N160 plants had significantly lower parasitized larval number compared to N80. If the host plant quality grows with nitrogen fertilization, as has been proven for *Coleoptera* and *Hymenoptera*^24^, the larvae feeding on highly fertilized plants may have developed faster and consequently with a lesser time of exposure to parasitoids. Moreover, additional N could intensify plant toxin^92,93^ production, which in the case of *Brassica* are mainly glucosinolates (GLS)^18^. *Brassicaceae* glucosinolates deter generalist herbivores but not specialists^19^ such as *B. aeneus* ^91^*.* It has been found, however, that at high GLS concentrations even specialists cannot completely degrade toxic GLS hydrolytic products. This in turn can favor the infestation by parasitoids due to the weakened immune system of the host^80^. On the other hand, under the influence of toxins, weakened beetle larvae may be avoided by parasitoids in host selection^25^. In light of these contradicting results it is clear that further investigation is needed into the reasons why parasitoids avoid plants with a higher N fertilization.

All of the identified parasitoid species were also detected in a three-year field study in Estonia^41^. The latter confirms the setup of the current study was close to natural conditions in respect to parasitoid diversity, which didn’t significantly differ between N treatments. In Switzerland, despite a high parasitism rate at times (0–54%), parasitism caused only 1–2% of pollen beetle mortality in an oilseed rape field^48^. Parasitized larvae are palatable and vulnerable to diseases and weather events, most of the mortality was possibly caused by predators and other unspecified factors. In light of this, the current experiment provides important information about parasitism rates when carnivory and weather influences are excluded. Apparently due to low number of parental parasitoids we found only 9% parasitism rate where an average for Estonia is 17–34%^41^. Based in low parasitism rate in our experiment we can speculate than the prey from N80 and N100 plants was rather chosen by their quality than by general availability.

## Conclusions

Fertilized with nitrogen *Brassica napus* plants had higher yield compared to untreated, with no difference between fertilized (N80–N160) individuals. Under more severe pollen beetle attack compared to untreated plants, N160 oilseed rape reached similar yield values, potentially avoiding and/or compensating for the herbivory. Moderately fertilized groups N80 and N100 had the highest parasitoid loads on pollen beetle larvae, with potential contributions from the following factors:

1. The quality of host larvae was optimal since food plant N content was improved compared to unfertilized plants;
2. The presence of pollen beetles provoked an increase of infochemicals orienting parasitoids to the attacked plants. In particular, GLS compound emissions as a proportion of total emission increased.

More research is needed to test if pollen beetles and their parasitoids react to repellents and attractants revealed in current study.

## Supplementary Information

Supplementary Information 1.

Supplementary Information 2.

## Data Availability

The datasets used and/or analysed during the current study are partly available from Supplementary materials and the rest from corresponding author on reasonable request.
