# Supplementary material for: Pollen beetle offspring is more parasitized under moderate nitrogen fertilization of oilseed rape due to more attractive volatile signal
Source: Sci Rep. 2022 Aug 22;12:14294. doi: 10.1038/s41598-022-18030-0 (PMC9395338; doi:10.1038/s41598-022-18030-0)
Supplement: Supplementary file 1 — Supplementary Information 1. [file 41598_2022_18030_MOESM1_ESM.docx]

**Supplementary material**

**Table S1.** Study design, number of replicates, dates and places of collection of the experimental organisms.

| **Species** | **Experiment** | **Replicates number** | **Collection date** | **Field coordinates**  **(field type)** |
| --- | --- | --- | --- | --- |
| *Brassica napus* (variety ‘*Visby*’) | VOC | 23 | 02.05.2013 | 58°21'N 26°40'E (ORG) |
| *Brassica napus* (variety ‘*Rohan*’) | HS & VOC | 79 & 11 | 28.04.2014 | 58°41'N 26°68' E (CON) |
| *Brassicogethes aeneus* adults | HS | 240 | 12.05.2014 | 58°31'N 26°49' E (NCF) |
|  | VOC | 92  44 | 03–24.06.2013  21 & 27.05. 2014 | 58°43'N 26°62**'** E (CON)  58°41'N 26°68' E (CON) |
| Adults of *B. aeneus* parasitoids  (*Phradis* spp., *Tersilochus* spp. and *Diospilus* spp.) | HS | 26 | 04.06.2014 | 58°43'N 26°62**'** E (CON) |
| VOC – volatile organic compound measurements  HS – host selection experiment for beetles and their parasitoids  ORG – organic field (no synthetic fertilizer nor insecticide applied)  CON – conventional oilseed rape field  NCF – non cultivated field, blooming wild flowers (mainly *Barbarea vulgaris* R.Br. subsp. *arcuata* (Opiz ex J.Presl et C Presl] M.Loehr) | | | | |

**Table S2.** Average ± SE VOC emission rates (pmol m^-2^ s^-1^) of *Brassica napus* plants before and during exposure to the herbivore (H) *Brassicogethes aeneus* in 2013 under different N fertilization treatments (N0–N120)

| **No** | **Compound** | **2013** | | | | | |
| --- | --- | --- | --- | --- | --- | --- | --- |
|  |  | **N0** | **N20** | **N120** | **N0 H** | **N20 H** | **N120 H** |
|  | ***Geranyl diphosphate (GDP) pathway volatiles*** | | | | | | |
| 1 | $\alpha$-Pinene | 318±220 | 806±483 | 757±603 | 508±400 | 556±248 | 383±187 |
| 2 | 3-Carene | 319±181 | 393±156 | 178±122 | 438±324 | 287±137 | 189±81.0 |
| 3 | Camphene | 21.9±17.3 | 7.7±3.7 | 9.2±9.2 | 20.1±19.0 | 45.7±35.1 | 28.4±13.6 |
| 4 | Limonene | 128±41.1 | 288±80.2 | 131±64.9 | 384±160 | 230±82.8 | 326±244 |
|  | ***Geranylgeranyl diphosphate (GGDP) pathway volatiles*** | | | | | | |
| 5 | 6-Methyl-5-hepten-2-one | 10.8±10.8 | 99.6±50.8 | 16.3±16.3 | 50.8±49.0 | 119±76.0 | 67.7±43.3 |
| 6 | Geranyl acetone | 4.1±4.1 | 36.6±27.8 | 7.4±5.9 | 6.6±3.9 | 47.0±30.1 | 20.4±8.7 |
|  | ***Lipoxygenase (LOX) pathway volatiles*** | | | | | | |
| 7 | *(Z)-*3-Hexen-1-ol | 62.9±39.4 | 34.4±31.6 | 78.1±55.0 | 67.3±55.7 | 20.4±7.2 | 1.4±1.4 |
| 8 | 2-Ethylhexanal | 88.4±71.7 | 194±90.3 | 39.7±19.2 | 277±211 | 105±55.4 | 46.9±20.4 |
| 9 | 2-Pentanone | 1.2±1.0 | 65.8±32.2 | 51.9±25.0 | 15.4±8.4 | 28.7±10.4 | 16.1±9.2 |
| 10 | Hexanal | 59.1±39.1 | 491±292 | 502±400 | 788±556 | 274±83.3 | 132±80.0 |
| 11 | Hexane | 1,333±1,309 | 467±246 | 208±65.4 | 1,000±974 | 327±115 | 1,394±1,168 |
| 12 | Pentanal | 15.9±9.7 | 380±248 | 531±376 | 339±329 | 161±59 | 85.2±59.1 |
|  | ***Glucosinolate (GLS) pathway volatiles*** | |  |  |  |  |  |
| 13 | 2-Propenenitrile | - | 25.0±14.7 | 13.5±6.9 | 192±189 | 38.9±29.2 | 10.6±5.0 |
| 14 | Dimethyl disulfide | 28.1±26.1 | 4.7±3.1 | - | 17.6±9.0 | 4.2±3.7 | 0.5±0.5 |
| 15 | Isocyanatocyclohexane | - | 75.2±75.2 | 1.2±1.2 | 123±123 | 48.4±36.5 | 18.6±18.6 |
| 16 | Isothiocyanatocyclohexane | - | 53.8±46.0 | 32.9±32.9 | 55.3±55.3 | - | 12.8±12.8 |
| 17 | Tetramethylthiourea | - | 487±436 | 126±126 | 104±104 | 126±125 | 13.6±12.6 |
| 18 | Tetramethylurea | - | 313±226 | 133±83 | 1,535±1,535 | 337±271 | 23.3±18.4 |
|  | ***Shikimate (SHI) pathway volatiles*** | |  |  |  |  |  |
| 19 | Acetophenone | 6.9±6.9 | 7.4±4.3 | 19.1±9.8 | 23.0±12.6 | 11.8±3.6 | 9.9±5.5 |
| 20 | Benzaldehyde | 122±92.9 | 330±91 | 394±120 | 184±117 | 366±108 | 217±111 |
| 21 | Benzene | 107±90.9 | 115±37 | 114±27.5 | 153±146 | 99.2±29.0 | 305±220 |
| 22 | Benzothiazole | - | 133±117 | 49.9±30.0 | 47.2±41.0 | 10.7±6.0 | 18.7±14.3 |
| 23 | Phenol | 32.5±31.9 | 84.8±46.0 | 39.8±18.1 | 380±374 | 83.3±32.6 | 38.6±17.9 |
| 24 | Toluene | 16.1±16.1 | 97.7±45.4 | 118±52.4 | 53.9±47.3 | 59.7±20.9 | 91.2±27.3 |
|  | ***Short-chained (C_2_-C_3_) oxygenated volatiles*** | | | | | | |
| 25 | Acetaldehyde | 398±286 | 1,097±397 | 895±269 | 791±724 | 401±153 | 1,610±507 |
| 26 | Acetone | 174±147 | 1,325±571 | 1,093±338 | 171±118 | 689±242 | 1,713±538 |
| 27 | Ethanol | 2,455±1,320 | 2,019±738 | 703±306 | 1,079±438 | 1,822±704 | 1,361±898 |
|  | ***Long-chained (С_7_-C_10_) saturated aldehydes*** | | | | | | |
| 28 | Decanal | 158±133 | 164±57.4 | 110±58.5 | 211±98 | 161±61 | 244±167 |
| 29 | Heptanal | 62.8±29.5 | 338±148 | 316±255 | 375±298 | 83.6±26.6 | 133±83.9 |
| 30 | Nonanal | 187±90.9 | 502±203 | 409±191 | 601±187 | 261±75.2 | 339±168 |
| 31 | Octanal | 47.2±47.2 | 244±139 | 266±233 | 106±82.5 | 73.5±23.4 | 116±93.2 |

N0–N120—fertilization treatments (kg ha^-1^ N).

The number of replicates in each treatment was: N0 = 6, N20 = 10, N120 = 7.

**Table S3.** Average ± SE VOC emission rates (pmol m^-2^ s^-1^) of *Brassica napus* plants before and during exposure to the herbivore (H) *Brassicogethes aeneus* in 2014 under different N fertilization treatments (N0–N160)

| **No** | **Compound** | **2014** | | | | | | | | | | | |
| --- | --- | --- | --- | --- | --- | --- | --- | --- | --- | --- | --- | --- | --- |
|  |  | **N0** | **N80** | **N100** | **N160** | | **N0 H** | | **N80 H** | | | **N100 H** | **N160 H** |
|  | ***Geranyl diphosphate (GDP) pathway volatiles*** | | | |  | |  | |  | | |  |  |
| 1 | $\alpha$-Pinene | 409±220 | 181±167 | 68.3±54.8 | 53.6±27.7 | | - | | 151±149 | | | 334±328 | 13.7±13.7 |
| 2 | 3-Carene | 223±223 | 135±126 | 63.1±53.0 | 42.5±35.8 | | - | | 86.3±86.3 | | | 175±130 | 2.2±2.2 |
| 3 | Camphene | 6.6±6.6 | 0.8±0.8 | 13.5±13.5 | 2.3±1.6 | | - | | 2.2±2.2 | | | 8.5±8.5 | - |
| 4 | D-Limonene | 151±45.3 | 9.6±9.6 | - | 8.4±5.5 | | - | | 65.0±65.0 | | | - | 31.7±31.7 |
|  | ***Geranylgeranyl diphosphate (GGDP) pathway volatiles*** | | | | | |  | |  | | |  |  |
| 5 | 6-Methyl-5-hepten-2-one | 32.9±32.9 | 37.1±36.1 | 22.6±22.6 | 37.6±37.6 | | 104±104 | | 20.5±15.2 | | | 4.1±4.1 | 23.7±23.7 |
| 6 | Geranyl acetone | - | 10.7±10.7 | 15.5±15.5 | - | | 9.4±9.4 | | 2.3±2.3 | | | - | 5.9±5.9 |
|  | ***Lipoxygenase (LOX) pathway volatiles*** | | | | | | |  | |  | |  |  |
| 7 | *(Z)-*3-Hexen-1-ol | - | 2.8±2.8 | - | - | | - | | 2.5±2.5 | | | - | - |
| 8 | 2-Ethylhexanal | 134±134 | 10.6±7.7 | 194±141 | 178±78.0 | | 13.3±13.3 | | 28.5±28.4 | | | 1321±748 | 79.6±58.1 |
| 9 | 2-Pentanone | 3.4±3.4 | - | 63.3±27.9 | 4.6±2.5 | | 130±125 | | 29.9±19.3 | | | 36.6±21.9 | 8.0±5.8 |
| 10 | Hexanal | - | 7.9±7.9 | 60.6±35.7 | 4.0±2.3 | | 38.6±38.6 | | 46.7±42.5 | | | 16.2±9.2 | 3.3±2.7 |
| 11 | Hexane | 297±13.5 | 1,694±1,585 | 192±146 | 41.9±41.9 | | 52.5±52.5 | | - | | | 164±107 | 224±147 |
| 12 | Pentanal | 3.9±3.9 | 3.5±2.4 | 118±64.8 | 6.5±6.5 | | 69.4±69.4 | | 24.7±16.4 | | | 33.2±33.2 | 4.8±2.5 |
|  | ***Glucosinolate (GLS) pathway volatiles*** | |  |  |  | |  | |  | | |  |  |
| 13 | 2-Propenenitrile | - | - | 287±144 | 38.4±24.4 | | 1,396±1,396 | | 8.9±8.9 | | | 558±358 | 52.9±26.7 |
| 14 | Dimethyl disulfide | 105±105 | 1.1±1.1 | 35.6±22.1 | 16.2±9.0 | | 79.7±79.7 | | 7.9±7.9 | | | 11.6±11.6 | - |
| 15 | Isocyanatocyclohexane | 14.7±14.7 | - | 514±375 | 68.2±66.5 | | 8,456±8,456 | | - | | | 562±392 | 91.5±191.5 |
| 16 | Methanethiol | 46.6±46.6 | 5.4±5.4 | - | - | | 525±525 | | 1.3±1.3 | | | 146±76 | 9.1±9.1 |
| 17 | Tetramethylthiourea | 1,678±1,678 | - | 143±143 | 168±158 | 26,153±26,153 | | | | | 177±177 | 166±166 | 43.3±29.0 |
| 18 | Tetramethylurea | 2,724±2,724 | - | 416±216 | 294±148 | 14,172±14,172 | | | | | 280±280 | 1,084±589 | 159±159 |
|  | ***Shikimate (SHI) pathway volatiles*** | |  |  |  | |  | |  | | |  |  |
| 19 | Acetophenone | 12.5±12.5 | 3.8±3.8 | 10.6±7.1 | 8.6±4.4 | | 147±147 | | - | | | 40.8±23.1 | 2.7±2.7 |
| 20 | Benzaldehyde | 93.2±93.2 | 291±82.8 | 754±674 | 221±140 | | - | | 17.3±11.8 | | | 750±398 | 113±113 |
| 21 | Benzene | 133±133 | 1,016±1,016 | 146.3±38.2 | 115±59.9 | | 30.9±30.9 | | 83.1±83.1 | | | 170±87.8 | 121±73.7 |
| 22 | Benzothiazole | 2,280±2,280 | 1,766 ±1,759 | 305±182 | 5.9±5.9 | | - | | 1,344 ±1,344 | | | 1,042±1,042 | 13.2±13.2 |
| 23 | Phenol | 11.1±11.1 | - | - | - | | 364±364 | | 25.7±24.8 | | | - | 8.0±8.0 |
| 24 | Toluene | 119±5.6 | 44.6±44.6 | 92.3±32.9 | 34.9±15.5 | | 44.3±2.3 | | 81.3±81.3 | | | 113±40.1 | 52.6±14.5 |
|  | ***Short-chained (C_2_-C_3_) oxygenated volatiles*** | | | | | | | | | | | | |
| 25 | Acetaldehyde | 6,434±569 | 2,324±1,599 | 3,961±1,921 | 1,678±214 | | 488±488 | | 1,298±899 | | | 3,619±1,971 | 1,672±170 |
| 26 | Acetone | 2,214±2,214 | 719±719 | 4,242±2,238 | 1,308±231 | | 4,261±3,719 | | 444±444 | | | 4,089±2,161 | 1,304±366 |
| 27 | Ethanol | 6,545±1,454 | 446±394 | 956±485 | 1,235±690 | | 7,920±3,416 | | 2,541±1,768 | | | 442±251 | 857±343 |
|  | ***Long-chained (С_7_-C_10_) saturated aldehydes*** | | | | | | | | | | | | |
| 28 | Decanal | 33.5±10.6 | 12.7±9.1 | 16.3±16.3 | - | | 121±60.8 | | 39.6±26.1 | | | 15.8±15.8 | - |
| 29 | Heptanal | - | 11.6±11.2 | 28.5±21.4 | - | | 10.4±10.4 | | 27.0±22.9 | | | 12.4±12.4 | - |
| 30 | Nonanal | 19.4±19.4 | 29.0±27.8 | - | - | | 74.2±74.2 | | 63.0±44.9 | | | 34.3±34.3 | - |
| 31 | Octanal | - | 17.3±17.3 | 9.7±6.6 | - | | 2.7±2.7 | | 24.5±19.5 | | | 20.1±20.1 | - |

N0–N160—fertilization treatments (kg ha^-1^ N).

The number of replicates in each treatment was: N0 = 2, N80 = 3, N100 = 3, N160 = 3.

(**a**) Pollen beetle release (**b**) Pollen beetle larvae collection


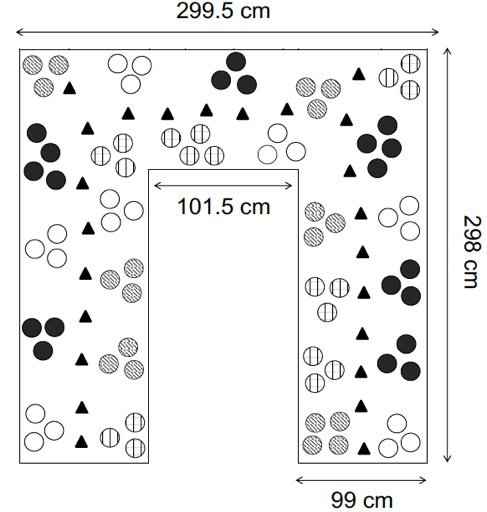


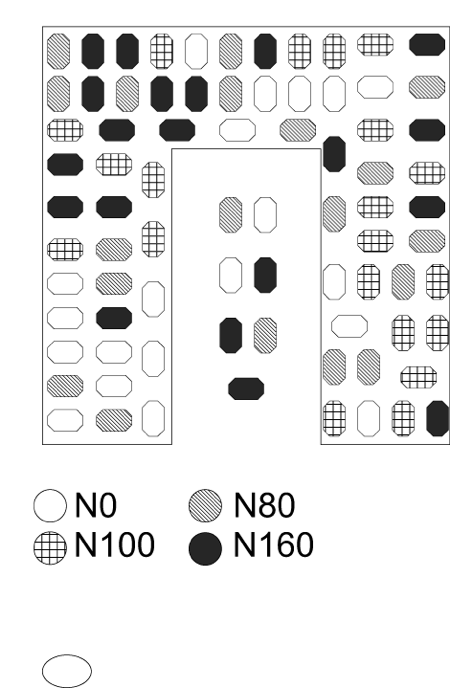


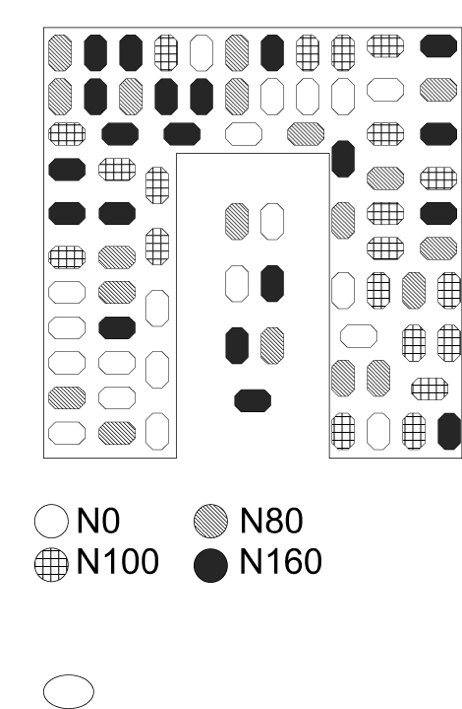
Nitrogen fertilization rate (kg ha^-1^)

**Figure S1.** Plant positions during Host Selection experiment in growth chamber. Different symbol fillings denote N fertilization rates. In (**a**), the circles represent a plant in groups of three-four individuals – with the same fertilization level. Small triangles stand for open lid plastic jars, each contained 10 pollen beetles on pest release day. Four weeks after beetle release (including one week after parasitoids release) plants were randomly distributed across the chamber (**b**), where each pot received a water trap (hexagon folium plate filled with water) on the ground.

| **VOC aggregation pattern of differently N fertilized *Brassica napus* plants (2013 and 2014) before and during infestation** | | |
| --- | --- | --- |
| **(a)** by pathways | **(b)** by compounds (detected >30% of plants) | |
| 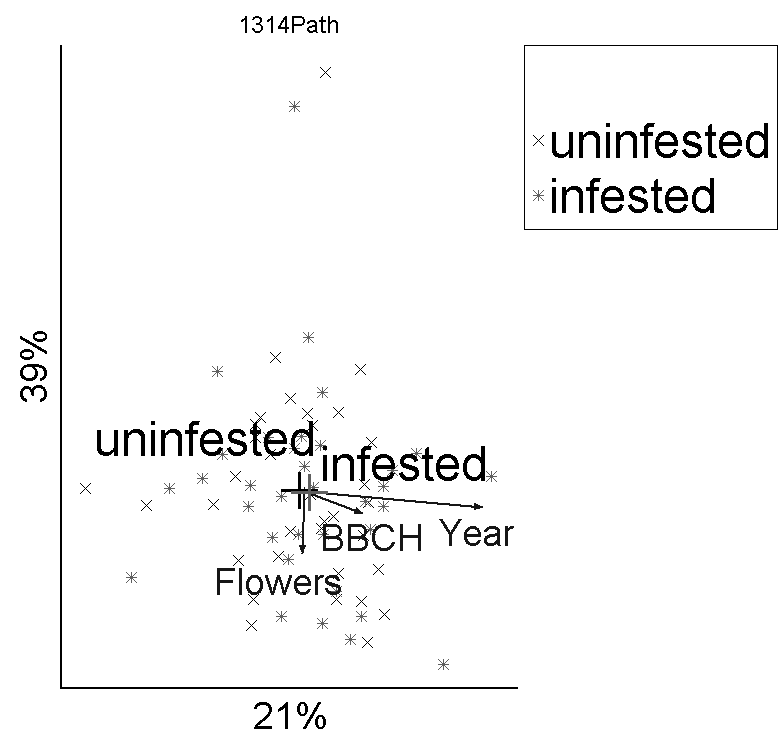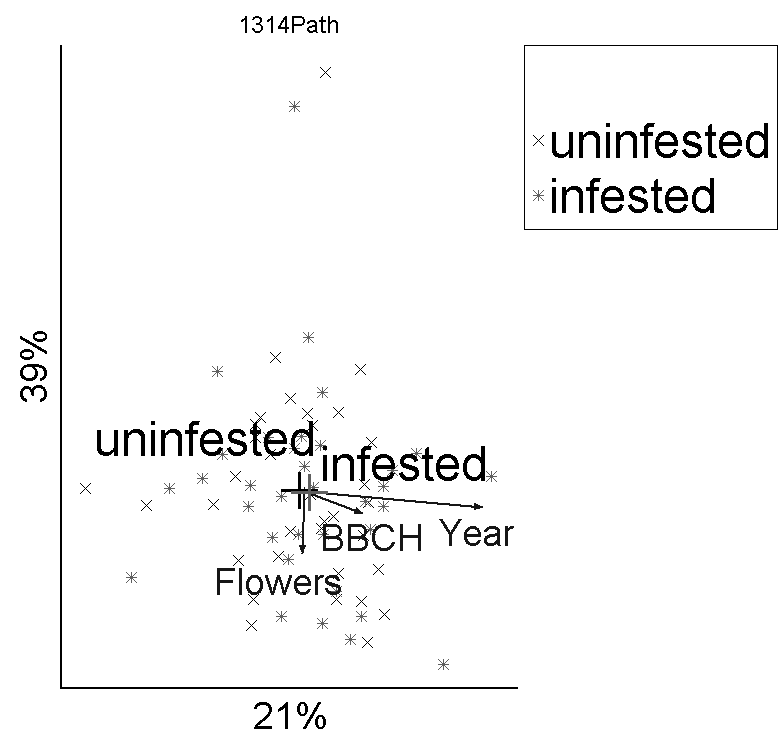 | 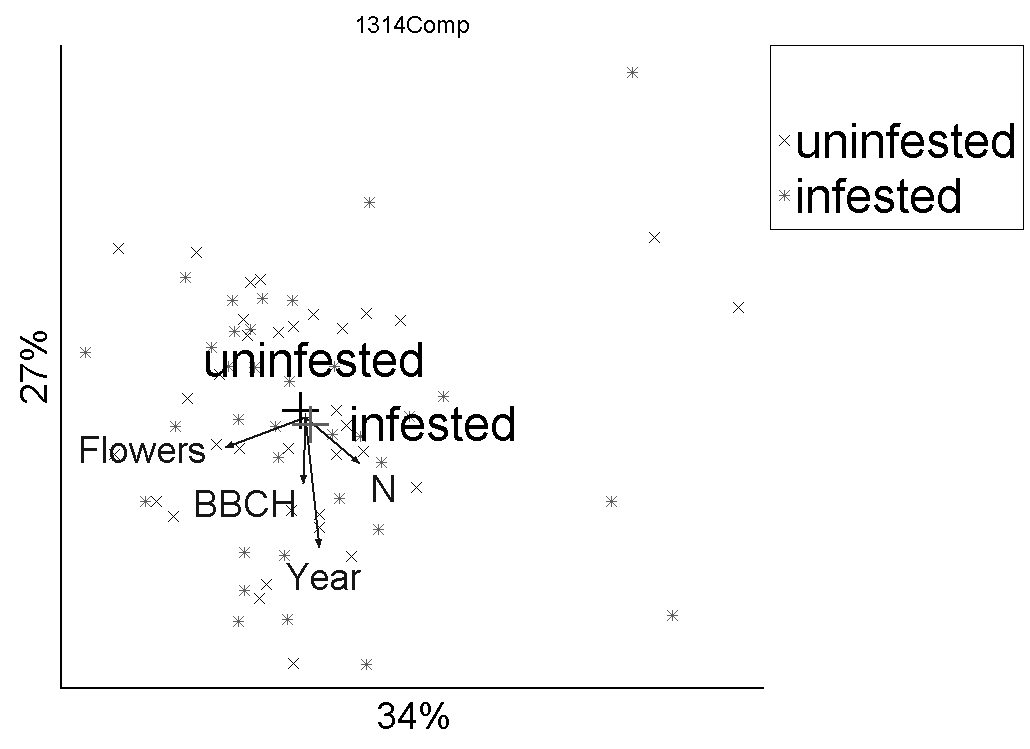 | |
|  |  |  |
| **Figure S2.** NMS ordination results of volatile organic compounds measured in 2013 and 2014 aggregated by pathways (**a**) and by compounds (**b**). N fertilization groups: 0, 20, 120 kg N ha^-1^ in 2013 and 0, 80, 100 and 160 kg N ha^-1^ in 2014. Plus sign is a centroid of differently fertilized plant samples based on VOC emission before and after *Brassicogethes aeneus* infestations. Vector shows correlation of plant smell with: growth stage (BBCH) and flower number in chamber during VOC collection (Flowers) as well as year of measurement (Year). All vectors *r^2^*>0.1 (scaling 200%), the length of which represent correlation strength. Axis percentage reflects importance to represented variance.  Not distinct N groups according to MRPP test: | |  |
|  |  |  |
| 1. N20_2013_ vs N120_2013_ (*A*=-0.013, *p*=1.0), N0_2013_ vs N20_2013_ (*A*=0.015, *p*=0.1), N20_2013_ vs N80_2014_ (*A*=0.019, *p*=0.1), N0_2013_ vs N120_2013_ (*A*=0.007, *p*=0.2), N80_2014_ vs N120_2013_ (*A*=0.015, *p*=0.2), N0_2013_ vs N80_2014_ (*A*=0.031, *p*=0.1), N0_2014_ vs N80_2014_ (*A*=0.020, *p*=0.2), N0_2014_ vs N100_2014_ (*A*=-0.009, *p*=0.5), N80_2014_ vs N100_2014_ (*A*=-0.016, *p*=0.6), N80_2014_ vs N160_2014_ (*A*=0.027, *p*=0.2), N100_2014_ vs N160_2014_ (*A*=0.073, *p*=0.1). | | |

1. N0_2013_ vs N20_2013_ (*A*=0.013, *p*=0.088), N20_2013_ vs N120_2013_ (*A*=0.007, *p*=0.138), N80_2014_ and N120_2013_ (*A*=0.018, *p*=0.072), N0_2013_ vs N80_2014_ (*A*=0.035, *p*=0.055), N0_2014_ vs N80_2014_ (*A*=0.022, *p*=0.218), N0_2014_ vs 100_2014_ (*A*=0.055, *p*=0.060), N80_2014_ vs N100_2014_ (*A*=0.008, *p*=0.320).
